# Optimizing Thermal-Elastic Properties of C/C–SiC Composites Using a Hybrid Approach and PSO Algorithm

**DOI:** 10.3390/ma9040222

**Published:** 2016-03-23

**Authors:** Yingjie Xu, Tian Gao

**Affiliations:** Engineering Simulation and Aerospace Computing (ESAC), Northwestern Polytechnical University, Xi’an 710072, China; gaotian_npu@163.com

**Keywords:** C/C–SiC composites, micromechanical modeling, BP neural network, particle swarm optimization algorithm, thermal-elastic properties

## Abstract

Carbon fiber-reinforced multi-layered pyrocarbon–silicon carbide matrix (C/C–SiC) composites are widely used in aerospace structures. The complicated spatial architecture and material heterogeneity of C/C–SiC composites constitute the challenge for tailoring their properties. Thus, discovering the intrinsic relations between the properties and the microstructures and sequentially optimizing the microstructures to obtain composites with the best performances becomes the key for practical applications. The objective of this work is to optimize the thermal-elastic properties of unidirectional C/C–SiC composites by controlling the multi-layered matrix thicknesses. A hybrid approach based on micromechanical modeling and back propagation (BP) neural network is proposed to predict the thermal-elastic properties of composites. Then, a particle swarm optimization (PSO) algorithm is interfaced with this hybrid model to achieve the optimal design for minimizing the coefficient of thermal expansion (CTE) of composites with the constraint of elastic modulus. Numerical examples demonstrate the effectiveness of the proposed hybrid model and optimization method.

## 1. Introduction

Carbon fiber-reinforced multi-layered pyrocarbon-silicon carbide matrix (C/C–SiC) composites exhibit attractive properties for thermal-structural applications, including low density, high strength, and high oxidation resistance. The multi-layered matrices consist in alternating sub-layers of pyrocarbon (PyC) and silicon carbide (SiC) [1,2]. The complicated spatial architecture and material heterogeneity of C/C–SiC composites constitute the challenge to understand their properties. Thus, discovering the intrinsic relations between the properties and the microstructures and sequentially optimizing the microstructures to obtain composites with the best possible performances becomes the key for practical applications of C/C–SiC composites.

The multi-layered matrices can be obtained by using the chemical vapor infiltration (CVI) process [3,4]. The controllable parameter in the CVI process is the layer thickness of each material. The layer thicknesses have to be properly controlled since the thickness variation of each layer affects the material microstructure and the effective properties of the composite as well. Many typical C/C–SiC composite components in aerospace engineering would be loaded in high temperature environments over hundreds even thousands of hours [5]. In such environments, the primary concern is to use materials with low thermal expansion behaviors and large elastic modulus. Motivated by this situation, optimization of the thicknesses of matrix layers within microstructure to minimize the coefficient of thermal expansion (CTE) of the unidirectional C/C–SiC composites is proposed in this paper. The constraint is imposed on the allowable elastic modulus according to real applications.

The micromechanical modeling approach, which provides the overall behavior of the composite through a finite element analysis of a unit cell model [6,7], is applied to obtain the CTE [8] and elastic modulus [9] of composites. The advantage of this approach is not only in obtaining global properties of the composite but also the behaviors that can be related to the composite microstructure. However, due to the complex multi-layered microstructure and large heterogeneity of multi-phase materials, a detailed unit cell finite element model of the unidirectional C/C–SiC composites involves a large number of elements. Generalization of the relationship between the microstructure and the overall properties of the composites using this finite element procedure is extremely difficult, especially in an optimization procedure. A new finite element mesh has to be rebuilt for each new situation and an iterative finite element analysis has to be carried out. This is extremely time consuming and computationally expensive. Thus, a hybrid approach is proposed in this paper by integrating the micromechanical model and artificial neural network for the identification of CTE and elastic modulus of the C/C–SiC composites. The artificial neural network has been extensively used in modeling composite material properties [10,11,12,13], especially for composite designing [14,15,16], as the relationship of the properties of the designed composite with its design parameters is very difficult to be represented as an explicit mathematical model. However, there is a lack of studies on applying the neural network in predicting the properties of C/C–SiC composites.

The nonlinear and non-differentiable nature of the presented optimization problem induces difficulty in using classical deterministic approaches for solutions. To solve this nonlinear optimization problem, a particle swarm optimization (PSO) algorithm [17,18] is used. The PSO algorithm belongs to the category of swarm intelligence techniques. It has only a small number of parameters that need to be adjusted, and is easy to implement. Although the PSO algorithm has been applied to a wide range of engineering problems in the literature [19,20,21,22], few applications to C/C–SiC composites are known.

In this study, a hybrid approach integrating the micromechanical model and artificial neural network is firstly proposed for the identification of CTE and elastic modulus of the unidirectional C/C–SiC composites. Predictions are compared with the results of a micromechanical model to assess the predictive capability of the proposed hybrid approach. The comparison shows that the forecast errors of the hybrid approach are inside the range of the relative fluctuations of testing samples. Although the neural network predictions partly agree with the micromechanical model, it is essential to improve the current neural network model in the future for an enhanced predicting capability. Then, a modified PSO algorithm is interfaced with the hybrid predictive model to minimize the CTE of a unidirectional C/C–SiC composite with six layers of alternating PyC and SiC matrix. The design variables are the thicknesses of matrix layers within the microstructure, and a constraint is imposed on the allowable elastic modulus. The classical PSO algorithm is modified to satisfy the constraints and the variable limits. The multi-stage penalty function method is adopted within PSO to satisfy the constraints, and the Harmony Search algorithm is used to deal with the particles that fly outside the variable boundaries.

## 2. Micromechanical Model of the Unidirectional C/C–SiC Composites

### 2.1. Unit Cell Model

**Figure 1:**
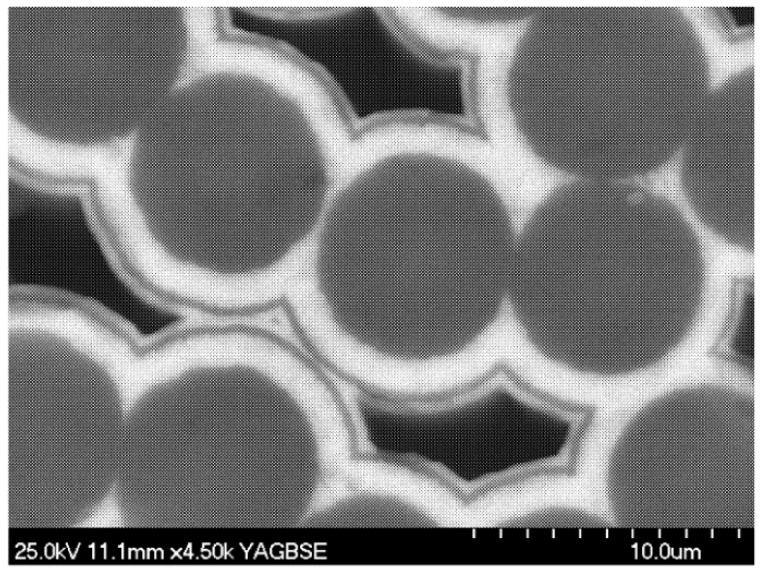
Scanning electron microscopy (SEM) photograph of a carbon fiber-reinforced multi-layered pyrocarbon-silicon carbide matrix (C/C–SiC) composite [23].

The architecture of the preform of unidirectional C/C–SiC composite consists of closely arranged fibers. The multi-layered PyC and SiC matrices are infiltrated within the porous fiber preforms by CVI process. Figure 1 shows the scanning electron microscope (SEM) photograph of a C/C–SiC composite [23], with the matrix consisting of alternate SiC (white color) and PyC (black color). It is clearly observed that the multi-layered matrices are distributed around the fibers. In addition, the pores are usually generated between adjacent fibers due to incomplete infiltration.

For the CVI-processed composites, the research of Chateau and Gélébart [24] has indicated that the residual pores after CVI process have an important influence on the mechanical behavior of composites. Thus, for an accurate simulation of the material behavior, one must carefully introduce these manufacturing flaws in a computing scheme. However, in the present study, a highly idealized unit cell model is employed. Our purpose is to use this idealized model to develop a numerical scheme in an efficient manner for optimizing the thermal-elastic properties of composites. Thus, the presented research in this paper puts more emphasis on creating a validated and expandable optimization scheme. However, it should be noted that for an accurate microstructure design of the C/C–SiC composites, the pores and fiber positions must be carefully captured and modeled. To do this, the X-ray micro-computed tomography is an effective tool that has shown its applicability in the work of Chateau and Gélébart [24]. In our future study, for a high-quality optimization of C/C–SiC composites, a more real unit cell including the heterogeneous pore and fiber distributions would be carefully modeled.

**Figure 2:**
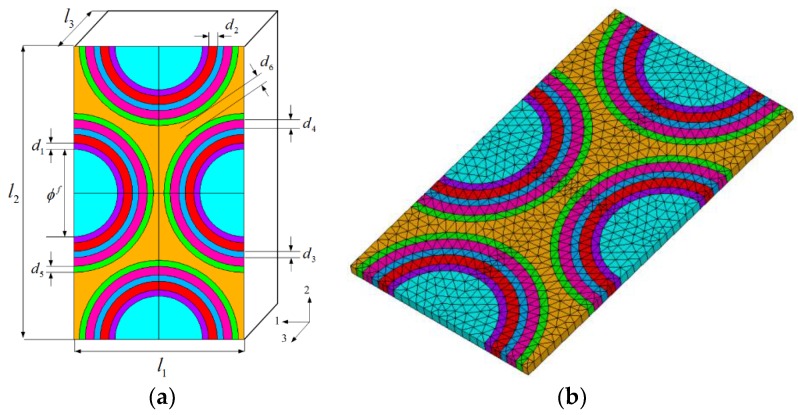
Unit cell of the unidirectional C/C–SiC composites: (**a**) Geometrical model; (**b**) Finite element model.

In this paper, a unidirectional C/C–SiC composite with six layers of alternating PyC and SiC is considered as a case study. A geometrical model of the unit cell is displayed in Figure 2a. Characteristic geometric parameters of the unit cell are given: φ*^f^* is fiber diameter and *d_1_*–*d_6_* are thicknesses of the matrix layers. The six layers of matrices are alternating PyC and SiC material layers (denoted as PyC/SiC/PyC/SiC/PyC/SiC). The unit cell model is then meshed using the 3-D twenty-node, thermal-structural coupled element (SOLID 96) of ANSYS finite element software, as depicted in Figure 2b.

### 2.2. Computation of the Elastic Modulus

In this study, a strain energy-based finite element approach is applied to evaluate effective elastic properties. It is assumed that each unit cell in the composites has the same deformation mode and that there is no separation or overlap between neighboring unit cells. Therefore, the periodic boundary conditions (PBC) [25,26,27] must be applied to the unit cell model. The PBC can be applied using nodes coupling (CP) and constraint equations (CE) defining in ANSYS. Then, the explicit formulations between the stiffness matrix coefficients and the strain energy of the unit cell model under specific loadings are derived. The detailed description of this method can be found in [28,29]. Here, only a basic introduction is presented.

In the elastic regime, the macroscopic behaviors of the unit cell can be characterized by the effective stress tensor $\bar{\sigma }$ and strain tensor $\bar{\varepsilon }$ over the homogeneous equivalent model. They are interrelated by the effective, also termed homogenized, stiffness matrix C*^H^*.

(1) $$\bar{\sigma }=C^{H}\bar{\varepsilon }$$

where $\bar{\sigma }=1/V\int_{\Omega }\sigma d\Omega$, $\bar{\varepsilon }=1/V\int_{\Omega }\varepsilon d\Omega$, and *V* is the volume of the unit cell.

Consider the case of 3-D orthotropic materials; Equation (1) corresponds to

(2) $$\left[\begin{array}{l} {\bar{\sigma }}_{11}\\ {\bar{\sigma }}_{22}\\ {\bar{\sigma }}_{33}\\ {\bar{\sigma }}_{12}\\ {\bar{\sigma }}_{23}\\ {\bar{\sigma }}_{31} \end{array}\right]=\left[\begin{array}{cccccc} C_{1111}^{H} & C_{1122}^{H} & C_{1133}^{H} & 0 & 0 & 0\\ C_{1122}^{H} & C_{2222}^{H} & C_{2233}^{H} & 0 & 0 & 0\\ C_{1133}^{H} & C_{2233}^{H} & C_{3333}^{H} & 0 & 0 & 0\\ 0 & 0 & 0 & C_{1212}^{H} & 0 & 0\\ 0 & 0 & 0 & 0 & C_{2323}^{H} & 0\\ 0 & 0 & 0 & 0 & 0 & C_{3131}^{H} \end{array}\right]\left[\begin{array}{l} {\bar{\varepsilon }}_{11}\\ {\bar{\varepsilon }}_{22}\\ {\bar{\varepsilon }}_{33}\\ {\bar{\varepsilon }}_{12}\\ {\bar{\varepsilon }}_{23}\\ {\bar{\varepsilon }}_{31} \end{array}\right]$$

The strain energy related to the microstructure is equal to:

(3) $$\begin{array}{l} E=\int_{\Omega }\frac{1}{2}\left(\sigma_{11}\varepsilon_{11}+\sigma_{22}\varepsilon_{22}+\sigma_{33}\varepsilon_{33}+\sigma_{12}\varepsilon_{12}+\sigma_{23}\varepsilon_{23}+\sigma_{31}\varepsilon_{31}\right)d\Omega \\ \;=\frac{1}{2}\left({\bar{\sigma }}_{11}{\bar{\varepsilon }}_{11}+{\bar{\sigma }}_{22}{\bar{\varepsilon }}_{22}+{\bar{\sigma }}_{33}{\bar{\varepsilon }}_{33}+{\bar{\sigma }}_{12}{\bar{\varepsilon }}_{12}+{\bar{\sigma }}_{23}{\bar{\varepsilon }}_{23}+{\bar{\sigma }}_{31}{\bar{\varepsilon }}_{31}\right)V \end{array}$$

With the help of specific loadings, the combination of Equation (2) and Equation (3) can be used to deduce the effective stiffness matrix C*^H^* for the unit cell. Suppose a unit initial strain is imposed in the y1 direction; *i.e.*, $\overset{-(1)}{\varepsilon }={\left(1\;0\;0\;0\;0\;0\right)}^{T}$. Note that the superscript (1) represents the first load case. The corresponding average stress is then obtained by Equation (2):

(4) $$\overset{-(1)}{\sigma }={\left(C_{1111}^{H}\;C_{1122}^{H}\;C_{1133}^{H}\;0\;0\;0\right)}^{T}$$

By replacing $\overset{-(1)}{\sigma }$ and $\overset{-(1)}{\varepsilon }$ into Equation (3), one obtains the following expression of the strain energy:

(5) $$E^{\left(1\right)}=\frac{1}{2}\overset{-(1)}{\sigma }\overset{-(1)}{\varepsilon }V=\frac{1}{2}C_{1111}^{H}V$$

The matrix coefficient $C_{1111}^{H}$ can be derived:

(6) $$C_{1111}^{H}=2E^{\left(1\right)}/V$$

In the same way, demonstrations can be made for other coefficients, and all the results are summarized in Table 1. The elastic properties can be derived by inversing the elastic matrix. In practice, the considered unit cell will be discretized into a finite element model on which the initial strain will be imposed to evaluate the strain energy.

### 2.3. Computation of the CTE

Here, the CTE of the composites is determined by finite element computation of the unit cell with specific structural and thermal loadings [30]. As shown in Figure 1a, along the planes *x*_1_ = 0, *x*_2_ = 0, and *x*_3_ = 0, the model is restricted to move in the *x*_1_, *x*_2_, and *x*_3_ directions. Planes *x*_1_ = *l*_1_, *x*_2_ = *l*_2_, and *x*_3_ = *l*_3_ are free to move but have to remain planar in a parallel way to preserve the compatibility with adjacent cells. Suppose the deformation of the unit cell is caused by a temperature rise of Δ*T*. During the deformation, *x_i_* = *l_i_* becomes *x_i_* = *l_i_* + Δ*l_i_*, and the displacement, Δ*l_i_*, can be determined from the finite element analysis. The CTE in direction *i* then corresponds to

(7) $$\alpha_{i}=\frac{\Delta l_{i}}{l_{i}\Delta T}$$

### 2.4. Experiment

To test the accuracy of the micromechanical model, three samples of the unidirectional C/C–SiC composites with different layer thicknesses were fabricated. The fiber preforms were close-packed 1K T-300 carbon fiber yearns from Nippon Toray (Tokyo, Japan). The multi-layered PyC and SiC matrix were deposited by CVI process using butane and methyltrichlorosilane (MTS) as the reactive materials in School of Material, Northwestern Polytechnical University, Xi’an, PR China. The infiltration condition of PyC was: temperature 960 °C, pressure 5 KPa, Ar flow 200 mL/min, butane flow 15 mL/min. The infiltration condition of SiC was: temperature 1000 °C, pressure 5 KPa, H_2_ flow 350 mL/min, Ar flow 350 mL/min, and the molar ratio of H_2_ and MTS of 10. Different layer thicknesses are obtained by controlling the infiltration time. The detailed illustration for the above process can be found in [3]. Elastic constants and CTEs of each material phase were taken from [3] and listed in Table 2. In this study, three groups of layers thicknesses were designed by controlling the infiltration time and are listed in Table 3. The fiber volume fractions of three samples were 19.7%, 19.7%, and 19.1%, respectively. Here, it should be noted that there indeed exists discrepancy between the designed thickness and the measured thickness, due to the complexity of the CVI process. However, since the purpose of this experiment is to verify the micromechanical model in an efficient manner, the discrepancy between the designed and true thickness values is neglected to simplify the experiment implementation (*i.e.*, avoid the complex measurement of layer thicknesses in the SEM microphotographs).

Uniaxial tensile tests were conducted at room temperature to obtain the longitudinal tensile modulus of the composites. Quasi-static tension tests were performed on a DNS-100 electronic universal testing system (CIMACH, Changchun, China). To measure the longitudinal and transverse CTEs of composites, DIL 402C dilatometer made by NETZSCH Company (Selb, Germany) was employed. Predictions based on the micromechanical model were compared with experimental data. The diameter of T-300 carbon fiber is 7.0 μm. The modulus and CTEs obtained in the previous tests for composite samples (denoted as A, B, and C) with different layer thicknesses, listed in Table 3, were chosen for comparison.

Table 3 shows the comparison of measured and predicted modulus and CTEs for the various composite samples. It can be seen that the predicted results coincide well with the experimental data. The comparative results may highlight the predictive capacity of the proposed micromechanical model for predicting the elastic modulus and CTEs of unidirectional C/C–SiC composite. However, it should be pointed out that because rudimental cavities generated within the composite for the emission of large infiltrated by-products during the infiltration are not considered in this paper; thus, the modulus and CTEs computed numerically by the present model are larger than the experimental results.

## 3. Optimization Problem

In this paper, a unidirectional C/C–SiC composite consisting of six layers of matrices made up of alternate PyC and SiC is used as a case study. In high temperature environments, one of the common requirements is to use C/C–SiC composites with low thermal expansion behaviors and high elastic modulus. Thus, the objective of this study is to minimize the CTE of composites with elastic modulus constraints. The optimization problems given in the present study include two cases, which include, respectively, the minimization of the longitudinal and transverse CTEs. A constant fiber volume fraction of 30% is defined for the composites. Therefore, an equality constraint is imposed on the sum of the thicknesses of matrix layers. Note that in order to simplify the programming implementation, this equality constraint is transferred to inequality constraints, as illustrated in Equations (8) and (9) (Δ = 0.01). *D*_0_ is a constant derived from the fiber volume fraction. In this study, *D*_0_ is equal to 5.168 for a 30% fiber volume fraction. In addition, since the load-bearing capability of C/C–SiC composite structures in industrial applications is primarily related to the tensile modulus *E*_33_, another constraint is imposed on the allowable value of *E*_33_ according to the real applications.

Mathematically, the optimization problems can be formulated as follows:

(8) $$\begin{array}{l} Minimize\;\alpha_{33}\left(X\right)\;\\ X=\left(d_{1},\;d_{2},\;...,\;d_{6}\right)\\ 0.2\leq d_{i}\leq 1.0,\;1\leq i\leq 6\\ Subject\;to\left\{ \begin{array}{l} 200.0-E_{33}\left(X\right)\;\leq 0\\ \;d_{6}+2\sum_{i=1}^{5}d_{i}-\left(D_{0}+\Delta \right)\leq 0\\ \;\left(D_{0}-\Delta \right)-d_{6}-2\sum_{i=1}^{5}d_{i}\leq 0 \end{array}\right.\; \end{array}$$

(9) $$\begin{array}{l} Minimize\;\alpha_{11}\left(X\right)\;\\ X=\left(d_{1},\;d_{2},\;...,\;d_{6}\right)\\ 0.2\leq d_{i}\leq 1.0,\;1\leq i\leq 6\\ Subject\;to\left\{ \begin{array}{l} 200.0-E_{33}\left(X\right)\;\leq 0\\ \;d_{6}+2\sum_{i=1}^{5}d_{i}-\left(D_{0}+\Delta \right)\leq 0\\ \;\left(D_{0}-\Delta \right)-d_{6}-2\sum_{i=1}^{5}d_{i}\leq 0 \end{array}\right. \end{array}$$

The design variables are the thicknesses of the matrix layers. The fiber diameter φ*^f^* is 7.0 μm. Elastic constants and CTEs of each material phase are listed in Table 2. The upper bound of matrix layer thickness is 1.0 μm. The lower bound of thickness of matrix is set to 0.2 μm for reducing the complexity of the fabrication process.

## 4. Back Propagation (BP) Neural Network Model

The proposed micromechanical modeling approach has shown favorable predicting capability for the thermal-elastic properties of C/C–SiC composites. However, a high computational cost would be induced due to the large number of elements of the complex multi-layered microstructure. Especially in an optimization procedure, for each new situation, a finite element mesh has to be rebuilt and an iterative finite element analysis has to be carried out. This is extremely time consuming and computationally expensive. The most important benefit of an artificial neural network is the high computing efficiency. Therefore, in this study a hybrid approach integrating the micromechanical model and an artificial neural network is proposed for the determination of the CTE and elastic modulus of the C/C–SiC composites.

The BP neural network has the powerful ability of non-linear interpolation to obtain the mathematical mapping reflecting the internal law of the experimental data. In this study, a four-layer BP neural network containing one input layer, two hidden layers and one output layer is developed to construct the mapping between the layer thicknesses and the thermal-elastic properties of unidirectional C/C–SiC composites. Every neural network has exactly one input layer and one output layer. So we only need to determine the number of hidden layers. Heaton [31] summarized the capabilities of neural network architectures with various hidden layers: the hidden layer is not needed if the function is linearly separable; one hidden layer can approximate any function that contains a continuous mapping from one finite space to another; two hidden layers can represent an arbitrary decision boundary to arbitrary accuracy with rational activation functions and can approximate any smooth mapping to any accuracy. Therefore, two hidden layers are used in this study. Although two hidden layers increases the computational cost, their capability of representing functions with any kind of shape provides a promising tool for our further study.

**Figure 3:**
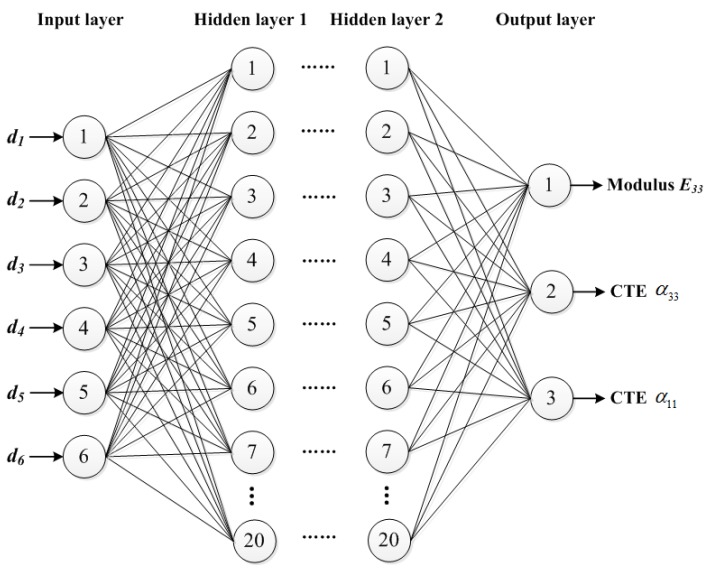
Back Propagation (BP) neural network architecture used in this study.

Figure 3 illustrates the BP neural network architecture used in this study. The network contains three parts: one input layer having six neurons related to the layer thicknesses; two hidden layers with 20 neurons for each one, and one output layer having three neurons representing the transverse and longitudinal CTEs α_11_ and α_33_ and the modulus *E*_33_. There are many general methods for determining the number of neurons in the hidden layers. However, these rules just provide a starting point for users to consider. For the problem considered in this study, one of the commonly-used rules gives an approximate range for the number of neurons in a hidden layer:

(10) $$n=\sqrt{n_{i}+n_{o}}+\alpha$$

where *n* is the number of neurons in the hidden layer, *n_i_* is the number of neurons in the input layer, *n_o_* is the number of neurons in the output layer, α is a constant from 1 to 10. According to the empirical equation, in this study the number of neurons in the hidden layer is between 4 and 13. Considering a BP neural network with more neurons in the hidden layer can give a higher precision solution [32]; the number of neurons in the hidden layer is finally selected as 20 in this paper.

In the network, the total input, *in_j_*, received by the *j*th neuron in the hidden layer from all of the neurons in the preceding layer is:

(11) $$in_{j}=\sum_{j=0}^{N}\omega_{ij}x_{i}$$

where *N* is the number of inputs to the *j*th neuron in the hidden layer, *x_i_* is the input from the *i*th neuron in the preceding layer, and *w_ij_* is the connection weight from the *i*th neuron in the forward layer to the *j*th neuron in the hidden layer.

The neuron then processes the input through a transfer function *f_s_* as below to produces its output *out_j_*:

(12) $$out_{j}=f_{s}\left(in_{j}\right)=\frac{1-e^{-in_{j}}}{1+e^{-in_{j}}}$$

Before the above BP neural network system can be used to predict the thermal-elastic properties of the composite, it must be trained by the data obtained from the micromechanical model. The connection weight *w_ij_* will be calculated by minimizing the error between the predictive value and the actual value during the training process. Details about the training process will be discussed in the following section.

### 4.1. Generation of Training Data

In this study, the training data are obtained from the micromechanical computations. In order to reflect the inner relationship between the thermal-elastic properties and the matrix layer thicknesses, a full factorial experimental design is no doubt an excellent idea. However, the full factorial experimental design means that a large number of computations (15,625 in this study) should be taken, which will obviously consume much time. Therefore, the Taguchi orthogonal array [33] which suggests using less simulation to find out the relationship between parameters is employed in this study. 25 samples designed by the L25 orthogonal array as well as another 35 samples randomly generated by computer, 60 samples in sum, are used to train the designed network.

### 4.2. Neural Network Training

During the training process, the connection weights should be calculated by minimizing the mean square error between network predictions and training data. Equation (13) [31] is used to update the connection weights iteratively. At the beginning of the training, the weights are given at random, and then they are iteratively updated until convergence to the certain values by using the gradient descent method.

(13) $$\begin{array}{l} \omega_{ij}^{new}=\omega_{ij}^{old}+\Delta \omega_{ij}\\ \Delta \omega_{ij}=-\eta \frac{\partial E}{\partial \omega_{ij}}out_{j} \end{array}$$

where *E* is the mean square error and is set as 1 × 10^−4^. η is the learning rate parameter controlling the stability and rate of convergence of the network, which is usually a constant between 0 and 1 and is chosen to be 0.01 in this study. Totally, the number of the connection weights to be identified is 580.

**Figure 4:**
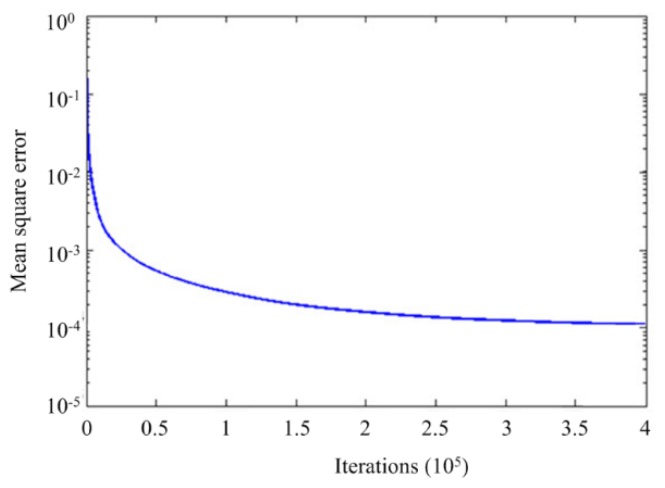
Training process of the network.

The training process takes about 1200 s of CPU time on HP personal workstation for 4.0 × 10^5^ training iterations. Figure 4 gives the variation curve of the mean square error with the iteration of connection weights (according to Equation (13)). It can be observed that with the updating of the connection weights, the mean square error has been gradually declined and converged to 1 × 10^−4^. The mathematic mapping between the layer thicknesses and the CTE and elastic modulus is then stored in the trained net. The mathematic function can be expressed as:

(14) $$S\left(i\right)=f_{l}\left(\sum w_{3}f_{s}\left(\sum w_{2}f_{s}\left(\sum w_{1}X\right)\right)\right)$$

where *S*(*i*) (*i* = 1, 2, 3) represents the CTE and elastic modulus; ***X*** = [*x_1_*, *x_2_*, *x_3_*, *x_4_*, *x_5_*, *x_6_*] is the vector consisting of the thickness values of six matrix layers; *f_l_* is the linear transfer function between hidden layer 2 and the output layer; *f_s_* is the transfer function between the input layer and hidden layer 1, as well as hidden layers 1 and 2; *w_1_*, *w_2_*, *w_3_* represent the connection weights between the input layer and hidden layer 1, hidden layer 1 and hidden layer 2, and hidden layer 2 and the output layer, respectively.

### 4.3. Neural Network Testing

In order to demonstrate the ability of a neural network system to generalize the training data, the neural network is used to estimate the modulus and CTEs of the input design parameter combination. Twenty groups of layer thicknesses randomly generated by computer (not used in the training process) are used in the testing and are listed in Table 4.

Table 5 shows the comparison between the neural network prediction and micromechanical computation. The forecast error is defined as:

(15) $$F^{e}=\left(S^{p}-S^{m}\right)/S^{m}$$

where *F^e^* represents the forecast error of the prediction system. *S^p^* is the result of neural network prediction, and *S^m^* stands for the result of micromechanical computation.

It is seen that the forecast errors of α_11_ and α_33_ are respectively inside the ranges of (−4.01%, 4.29%) and (−4.93%, 4.88%). Here, if the relative fluctuation of *S^m^* is defined as ($\left(S_{min}^{m}-\frac{S_{max}^{m}\;+\;S_{min}^{m}}{2}\right)/\frac{S_{max}^{m}\;+\;S_{min}^{m}}{2}$, $\left(S_{max}^{m}-\frac{S_{max}^{m}\;+\;S_{min}^{m}}{2}\right)/\frac{S_{max}^{m}\;+\;S_{min}^{m}}{2}$), we can easily obtain the relative fluctuations of α_11_ and α_33_ within the 20 groups of testing samples: (−10%, 10%) and (−11%, 11%). The above results clearly indicate that both the forecast errors of α_11_ and α_33_ fall in the range of fluctuations. Therefore we want to emphasize that although the neural network predictions partly agree with the micromechanical model, the improvement of the current neural network is still needed in future for an enhanced predicting capability. Possible refining approaches include supplementing adequate training samples with a wider range and optimizing the neural network. The running time of the prediction system is sharply decreased compared to that of the micromechanical analysis. The average running time of one micromechanical computation (including 12 finite element analyses) is about 2500 s and that of neural network prediction system is only about 0.001 s.

## 5. Particle Swarm Optimization Algorithm

In a PSO algorithm, each individual of the swarm is considered as a flying particle in the design space that has a position and a velocity. The particles remember the best position that they have seen during the flying. Members of a swarm remember the location where they had their best success and communicate good positions to each other, then update their own position and velocity based on these good positions as follows:

(16) $$V_{i}^{k+1}=\omega V_{i}^{k}+c_{1}r_{1}\left(P_{i}^{k}-X_{i}^{k}\right)+c_{2}r_{2}\left(P_{g}^{k}-X_{i}^{k}\right)$$

(17) $$X_{i}^{k+1}=X_{i}^{k}+V_{i}^{k+1}$$

where *V_i_* and *X_i_* represent the velocity and the position of the *i*th particle, respectively (the subscripts *k* and *k* + 1 refer to the recent and the next iterations, respectively). *P_i_* is the best previous position of the *i*th particle and *P_g_* is the best global position among all the particles in the swarm. ω is the inertia weight controlling the impact of the previous history of velocities on the current velocity and is set to 0.875 in this study. *c*_1_ and *c*_2_ are acceleration constants indicating the stochastic acceleration terms which pull each particle towards the best position attained by the particle or the best position attained by the swarm. In this work, *c*_1_ = 2 and *c*_2_ = 2 are chosen. *r*_1_ and *r*_2_ are two random numbers between 0 and 1.

Most optimization problems include the problem-specific constraints and the variable limits. For the present optimization, the problem-specific constraint is the elastic modulus, and the variable limits are design bounds of the thicknesses of matrix layers. If the particle flies out of the variable boundaries, the solution cannot be used even if the problem-specific constraint is satisfied, so it is essential to make sure that all of the particles fly inside the variable boundaries, and then to check whether they violate the problem-specific constraint.

### 5.1. Harmony Search Scheme: Handling the Variable Limits

**Figure 5:**
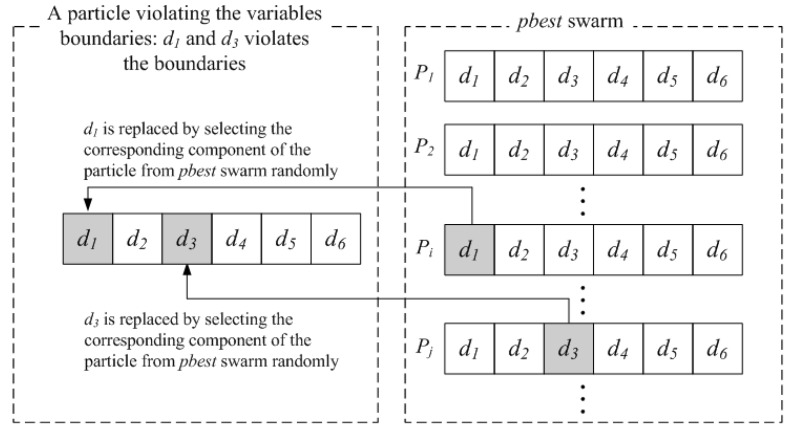
Illustration of the variable limits handling strategy.

A method introduced by Li *et al.* [34] dealing with the particles that fly outside the variable boundaries is used in the present study. This method is derived from the harmony search (HS) algorithm. In the HS algorithm, the harmony memory (HM) stores the feasible vectors, which are all in the feasible space. The harmony memory size determines how many vectors can be stored. A new vector is generated by selecting the components of different vectors randomly in the harmony memory. Undoubtedly, the new vector does not violate the variables boundaries. When it is generated, the harmony memory will be updated by accepting this new vector if it gets a better solution and deleting the worst vector. Similarly, the PSO algorithm stores the feasible and “good” vectors (particles) in the *p_best_* swarm, as does the harmony memory in the HS algorithm. Hence, the vector (particle) violating the variable boundaries can be generated randomly again by such a strategy: if any component of the current particle violates its corresponding boundary, then it will be replaced by selecting the corresponding component of the particle from *p_best_* swarm randomly. To highlight the presentation, a schematic diagram is given in Figure 5 to illustrate this strategy.

### 5.2. Penalty Functions Method: Handling the Problem-Specific Constraints

The most common method of handling the constraints is the use of a penalty function. The constrained problem is transformed to an unconstrained one, by penalizing the constraints and building a single objective function. Hence, the optimization problem becomes one of minimizing the objective function and the penalty together. In this paper, a non-stationary, multi-stage penalty function method implemented by Parsopoulos and Vrahatis [35] is adopted for constraint handling with PSO. The penalty function is

(18) $$F\left(X\right)=f\left(X\right)+h\left(k\right)H\left(X\right),\;X\in S\subset R^{n}$$

where *f(X)* is the original objective function to be optimized. *h(k)* is a penalty value which is modified according to the algorithm’s current iteration number *k* and is usually set to $h\left(k\right)=\sqrt{k}$. *H(X)* is a penalty factor defined as:

(19) $$H\left(X\right)=\sum_{i=1}^{m}\theta \left(q_{i}\left(X\right)\right)q_{i}{\left(X\right)}^{\gamma \left(q_{i}\left(X\right)\right)}$$

where *q_i_(X)* is a relative violated function of the constraints, which is defined as *q_i_(X)* = max {0, *g_i_(X)*} (Note that *g_i_(X)* is the constraint), $\theta \left(q_{i}\left(X\right)\right)$ is an assignment function, and $\gamma \left(q_{i}\left(X\right)\right)$ is the power of the penalty function. Regarding the [10], the following values are used for the penalty function:

If $q_{i}\left(X\right)<1$, then $\gamma \left(q_{i}\left(X\right)\right)=1$;
otherwise $\gamma \left(q_{i}\left(X\right)\right)=2$;
If $q_{i}\left(X\right)<0.001$, then $\theta \left(q_{i}\left(X\right)\right)=10$;
else if $q_{i}\left(X\right)\leq 0.1$, then $\theta \left(q_{i}\left(X\right)\right)=20$;
else if $q_{i}\left(X\right)\leq 1$, then $\theta \left(q_{i}\left(X\right)\right)=100$;
otherwise $\theta \left(q_{i}\left(X\right)\right)=300$.

## 6. Results

The optimization problems illustrated in Section 3 are implemented by using the proposed hybrid approach and PSO algorithm. For all the optimization problems, a population of 50 particles is used. The stopping criterion can be defined based on the number of iterations without an update in the best values of the swarm or the number of iterations the algorithm executes. Although the latter is not a real physical stopping criterion, it is quite easy in programming implementation and hence is widely used in PSO algorithms. In this work, the maximum number of iterations is limited to 100 and is adopted as the stopping criterion.

**Figure 6:**
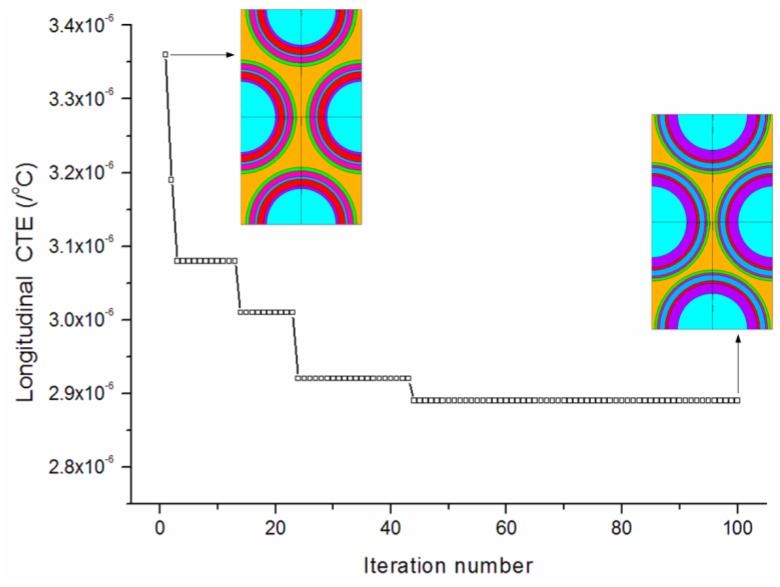
Convergence rates of the optimization procedure for minimizing the longitudinal CTE.

**Figure 7:**
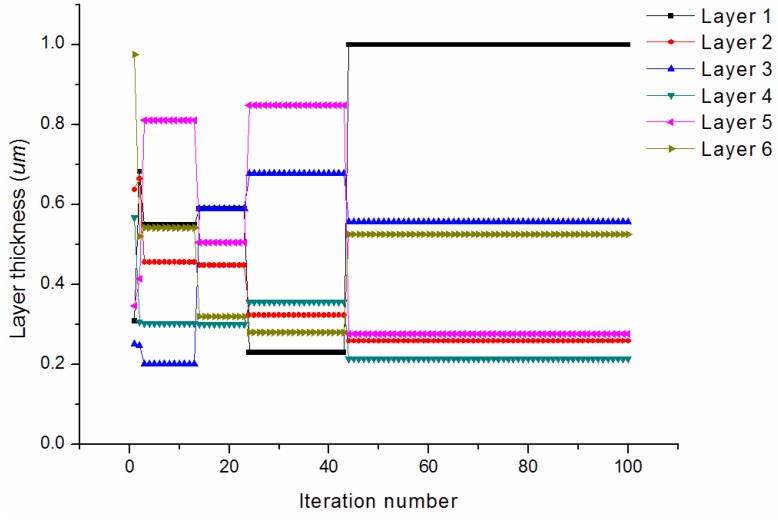
Convergence of the design variables for minimizing the longitudinal CTE.

Figure 6 provides the convergence rates of the optimization procedure for minimizing the longitudinal CTE. The algorithm achieves the best solution after about 50 iterations. The longitudinal CTE has been effectively reduced to 2.89 × 10^−6^/°C. The convergence of the design variables during the iterations is shown in Figure 7. It is observed that the thicknesses of the first (PyC) matrix layer increases to its higher bound. The third (PyC) and last (PyC) matrix layers both reach median values between the lower and higher bounds. The second (SiC), fourth (SiC), and fifth (PyC) matrix layers all iterate to values near the lower bound. The final optimized thicknesses are 0.999/0.259/0.557/0.215/0.276/0.525 μm.

**Figure 8:**
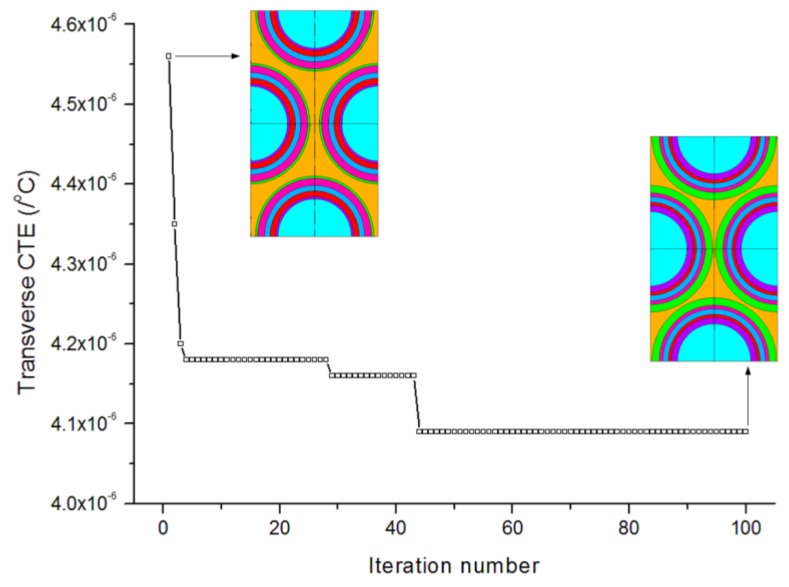
Convergence rates of the optimization procedure for minimizing the transverse CTE.

**Figure 9:**
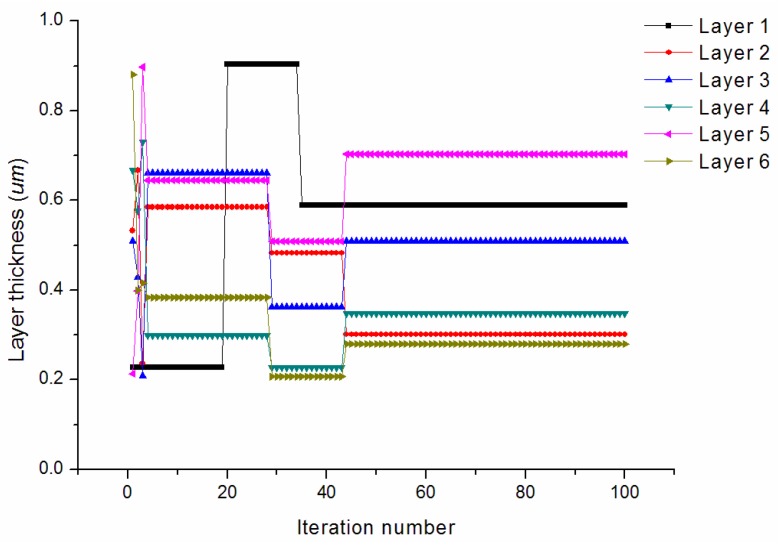
Convergence of the design variables for minimizing the transverse CTE.

Figure 8 provides the convergence rates of the optimization procedure for minimizing the transverse CTE. The algorithm achieves the best solution after about 80 iterations. The transverse CTE has been effectively reduced to 4.09 × 10^−6^/°C. The convergence of the design variables during the iterations is shown in Figure 9. It is observed that the thicknesses of the first (PyC) and third (PyC) matrix layers both iterate to median values between the lower and higher bounds. The second (SiC) and fifth (SiC) matrix layers iterate to values near the lower bound. The final optimized thicknesses are 0.589/0.301/0.510/0.349/0.703/0.279 μm.

## 7. Conclusions

In this study, optimal design of unidirectional C/C–SiC composites with respect to the thermal-elastic properties is obtained by the use of the non-gradient PSO algorithm. A hybrid methodology using a micromechanical model and a BP neural network is firstly proposed for predicting the elastic modulus and CTEs. Numerical results demonstrate its ability to find out the highly non-linear relationship between the multi-layers thicknesses and the CTEs and elastic moduli. However, it should be mentioned that the forecast errors of the presented neural network model are in the range of the relative fluctuations of testing samples. Therefore, although the neural network predictions partly agree with the micromechanical model, the improvement of the current neural network model is still needed in the future for an enhanced predicting capability.

Then, an optimization scheme which combines a PSO algorithm and the hybrid methodology was used to minimize the CTE of composites with the constraint of elastic modulus by designing the thicknesses of matrix layers. The minimization procedures of the longitudinal and transverse CTEs generate quite different thicknesses of matrix layers. The final optimized thicknesses are 0.999/0.259/0.557/0.215/0.276/0.525 μm for minimizing the longitudinal CTE, while for minimization of the transverse CTE the final optimized thicknesses are 0.589/0.301/0.510/0.349/0.703/0.279 μm.

Here, we want to mention that the emphasis of this work was to develop an effective optimization scheme for optimizing the thermal-elastic properties of unidirectional C/C–SiC composites. Numerical examples have shown the effectiveness of the proposed method. However, to obtain a variation law of the multi-layer thicknesses for the thermal-elastic design of C/C–SiC composites, more optimization cases would need to be investigated in our further studies.

## Figures and Tables

**Table 1:** Different loadings and the coefficients of elastic matrix.

Load Case	Loadings	Effective Coefficients of Elastic Matrix
1	$\overset{-(1)}{\varepsilon }={\left(1\;0\;0\;0\;0\;0\right)}^{T}$	$C_{1111}^{H}=2E^{\left(1\right)}/V$
2	$\overset{-(2)}{\varepsilon }={\left(0\;1\;0\;0\;0\;0\right)}^{T}$	$C_{2222}^{H}=2E^{\left(2\right)}/V$
3	$\overset{-(3)}{\varepsilon }={\left(0\;0\;1\;0\;0\;0\right)}^{T}$	$C_{3333}^{H}=2E^{\left(3\right)}/V$
4	$\overset{-(4)}{\varepsilon }={\left(0\;0\;0\;1\;0\;0\right)}^{T}$	$C_{1212}^{H}=2E^{\left(4\right)}/V$
5	$\overset{-(5)}{\varepsilon }={\left(0\;0\;0\;0\;1\;0\right)}^{T}$	$C_{2323}^{H}=2E^{\left(5\right)}/V$
6	$\overset{-(6)}{\varepsilon }={\left(0\;0\;0\;0\;0\;1\right)}^{T}$	$C_{3131}^{H}=2E^{\left(6\right)}/V$
7	$\overset{-(7)}{\varepsilon }={\left(1\;1\;0\;0\;0\;0\right)}^{T}$	$C_{1122}^{H}=E^{\left(7\right)}/V-C_{1111}^{H}/2-C_{2222}^{H}/2$
8	$\overset{-(8)}{\varepsilon }={\left(0\;1\;1\;0\;0\;0\right)}^{T}$	$C_{2233}^{H}=E^{\left(8\right)}/V-C_{2222}^{H}/2-C_{3333}^{H}/2$
9	$\overset{-(9)}{\varepsilon }={\left(1\;0\;1\;0\;0\;0\right)}^{T}$	$C_{1133}^{H}=E^{\left(9\right)}/V-C_{1111}^{H}/2-C_{3333}^{H}/2$

**Table 2:** Properties for each material phase.

Material Phase	E_11_ GPa	E_33_ GPa	G_12_ GPa	G_23_ GPa	*v*_12_	*v*_23_	α_11_ 10^−6^/°C	α_33_ 10^−6^/°C
T-300 carbon fiber	22	220	7.75	4.8	0.42	0.12	8.85	−0.7
pyrolytic carbon	12	30	2.0	4.3	0.4	0.12	1.8	–
silicon carbide	350	–	145.8	–	0.2	–	4.5	–

**Table 3:** Comparison of measured and predicted modulus and coefficients of thermal expansion (CTEs).

Sample	Layer Thickness (μm)						Modulus *E*_33_ (GPa)		CTE α_33_ (10^−6^/°C)		CTE α_11_ (10^−6^/°C)	
	*d_1_*	*d_2_*	*d_3_*	*d_4_*	*d_5_*	*d_6_*	Model	Test	Model	Test	Model	Test
A	0.5	1.0	0.5	1.0	0.5	1.0	256.2	226.1	3.753	3.309	4.356	3.893
B	0.5	0.5	0.5	1.0	1.0	1.0	225.5	200.1	3.646	3.235	4.242	3.847
C	1.0	0.5	1.0	0.5	1.0	0.5	175.1	155.8	3.421	3.021	4.009	3.630

**Table 4:** 20 groups of layer thicknesses used in the testing.

Nos.	Layer Thickness (μm)					
	*d_1_*	*d_2_*	*d_3_*	*d_4_*	*d_5_*	*d_6_*
1	0.2610	0.5662	0.2480	0.3919	0.9195	0.3899
2	0.4644	0.8772	0.2833	0.2048	0.2793	0.6328
3	0.4830	0.4023	0.4673	0.7826	0.4900	0.6968
4	0.5711	0.3550	0.9901	0.3457	0.3629	0.6663
5	0.7319	0.7946	0.5799	0.3282	0.7503	0.7479
6	0.9353	0.4803	0.8645	0.8642	0.6044	0.6487
7	0.8524	0.8761	0.7855	0.8279	0.2979	0.7361
8	0.9357	0.5894	0.7152	0.5864	0.2819	0.4140
9	0.7467	0.7546	0.2069	0.8498	0.8370	0.5739
10	0.7100	0.6951	0.9807	0.4282	0.9433	0.2059
11	0.7460	0.2228	0.2224	0.9292	0.3738	0.2794
12	0.7125	0.9604	0.9500	0.2565	0.9891	0.4261
13	0.8549	0.4947	0.8741	0.7923	0.8863	0.5873
14	0.3470	0.7968	0.8577	0.3749	0.9302	0.6073
15	0.8721	0.5665	0.3505	0.2041	0.4703	0.9845
16	0.8748	0.3878	0.8542	0.8348	0.5209	0.4124
17	0.9856	0.6024	0.3441	0.6426	0.7592	0.3716
18	0.9970	0.2374	0.4513	0.5714	0.9666	0.6571
19	0.8432	0.7456	0.8845	0.9966	0.4197	0.7483
20	0.2610	0.7432	0.4422	0.7643	0.6063	0.7591

**Table 5:** Comparison between the neural network prediction and micromechanical computation.

Nos.	CTE α_33_ (10^−5^/°C)			CTE α_11_ (10^−5^/°C)			Modulus *E*_33_ (GPa)		
	*S^p^*	*S^m^*	*F^e^* (%)	*S^p^*	*S^m^*	*F^e^* (%)	*S^p^*	*S^m^*	*F^e^* (%)
1	0.2978	0.3115	−4.40	0.4019	0.4163	−3.46	211.031	220.368	−4.24
2	0.3114	0.3218	−3.24	0.4637	0.4459	3.99	243.368	251.517	−3.24
3	0.3256	0.3390	−3.96	0.4520	0.4369	3.47	228.380	236.248	−3.33
4	0.3276	0.3164	3.54	0.4133	0.4306	−4.01	190.841	200.759	−4.94
5	0.3336	0.3451	−3.33	0.4057	0.4194	−3.25	202.234	209.252	−3.35
6	0.3711	0.3581	3.62	0.4091	0.4234	−3.36	215.198	207.463	3.73
7	0.3860	0.3681	4.88	0.4224	0.4358	−3.06	225.689	233.787	−3.46
8	0.3514	0.3392	3.58	0.4158	0.4296	−3.20	206.528	213.309	−3.18
9	0.3460	0.3584	−3.47	0.4345	0.4188	3.74	237.316	228.926	3.66
10	0.3461	0.3338	3.68	0.4014	0.3871	3.69	173.465	179.566	−3.40
11	0.3333	0.3226	3.31	0.4234	0.4380	−3.35	242.812	233.045	4.19
12	0.3560	0.3438	3.56	0.4065	0.3927	3.51	191.179	185.067	3.30
13	0.3684	0.3552	3.73	0.4260	0.4118	3.45	202.535	196.341	3.15
14	0.3534	0.3409	3.67	0.3931	0.4085	−3.78	211.295	203.589	3.78
15	0.3403	0.3283	3.64	0.4258	0.4412	−3.49	227.551	218.387	4.20
16	0.3343	0.3456	−3.27	0.4046	0.4213	−3.98	197.096	204.209	−3.48
17	0.3261	0.3430	−4.91	0.4280	0.4127	3.69	213.942	206.342	3.68
18	0.3196	0.3311	−3.47	0.4321	0.4163	3.80	193.728	187.862	3.12
19	0.3891	0.3715	4.76	0.4476	0.4319	3.62	236.370	228.074	3.64
20	0.3415	0.3592	−4.93	0.4475	0.4290	4.29	224.879	232.518	−3.29
